# Novelty Manipulations, Memory Performance, and Predictive Coding: the Role of Unexpectedness

**DOI:** 10.3389/fnhum.2020.00152

**Published:** 2020-04-29

**Authors:** Richárd Reichardt, Bertalan Polner, Péter Simor

**Affiliations:** ^1^Department of Cognitive Science, Budapest University of Technology and Economics, Budapest, Hungary; ^2^Institute of Psychology, Eötvös Lóránd University, Budapest, Hungary; ^3^Institute of Behavioural Sciences, Semmelweis University, Budapest, Hungary; ^4^UR2NF, Neuropsychology and Functional Neuroimaging Research Unit, CRCN—Center for Research in Cognition and Neurosciences and UNI—ULB Neurosciences Institute, Université Libre de Bruxelles (ULB), Brussels, Belgium

**Keywords:** memory, novelty, surprise, expectation, predictive coding

## Abstract

Novelty is central to the study of memory, but the wide range of experimental manipulations aimed to reveal its effects on learning produced inconsistent results. The novelty/encoding hypothesis suggests that novel information undergoes enhanced encoding and thus leads to benefits in memory, especially in recognition performance; however, recent studies cast doubts on this assumption. On the other hand, data from animal studies provided evidence on the robust effects of novelty manipulations on the neurophysiological correlates of memory processes. Conceptualizations and operationalizations of novelty are remarkably variable and were categorized into different subtypes, such as stimulus, context, associative or spatial novelty. Here, we summarize previous findings about the effects of novelty on memory and suggest that predictive coding theories provide a framework that could shed light on the differential influence of novelty manipulations on memory performance. In line with predictive coding theories, we emphasize the role of unexpectedness as a crucial property mediating the behavioral and neural effects of novelty manipulations.

## Introduction

Any notion to be explored by the scientific community needs to be defined unambiguously and novelty is not an exception. The so-called common sense or memory-based definition states that novelty is any aspect of a percept that is not already contained in the memory systems of the observer (Berlyne, 1960; Barto et al., 2013). Accordingly, any novelty experienced by the observer requires novel representations to be generated for that stimulus or event. The novelty/encoding hypothesis postulates that this newly created representation is more easily reactivated later, yielding enhanced recognition on the behavioral level (Tulving and Kroll, 1995).

The novelty/encoding hypothesis was partly based on the early findings of positron emission tomography (PET) studies showing that novel stimuli elicited activations in the hippocampal formation, the medial dorsal nucleus of the thalamus and the anterior and inferior parts of the cingulate cortex (Tulving et al., 1994, 1996). In a behavioral study inspired by these findings, participants had to learn lists containing words, some of which were familiarized before the experiment, while others were novel to the participants. In line with the novelty/encoding hypothesis, recognition rates for novel words were significantly higher than those for familiar words (Tulving and Kroll, 1995).

Nevertheless, the interpretation of the data was soon challenged—it was pointed out that hit rates for novel vs. familiar stimuli may have differed because the false alarm rate for familiar words was relatively higher, suggesting that the results may have stemmed from an interference effect within the familiar stimulus set (Dobbins et al., 1998). This interference-based explanation of the original findings, however, failed to completely refute the novelty/encoding hypothesis, as other studies still supported a memory-enhancing effect of novelty. For instance, a repetition-based study showed that the probability of recognition for the novel (and rare) stimuli was linked to the activation in prefrontal and temporal regions during encoding (Kirchhoff et al., 2000), while another study evidenced the novelty effect in a verbal recognition memory test (Kormi-Nouri et al., 2005).

Still, more recent comprehensive reviews tend to conclude that the novelty/encoding hypothesis is not unequivocally supported by empirical studies (Poppenk et al., 2010; Schomaker and Meeter, 2015). However, a parallel line of animal studies produced more consistent results. These inquiries revealed a clear association between novelty manipulations and electrophysiological and molecular markers of memory processes, such as dopaminergic modulation of long-term potentiation (LTP) in the hippocampus (Lisman and Grace, 2005). In these studies, the experimental animals were usually placed in a novel environment, while electrophysiological or neurochemical variables were registered (Floresco et al., 2001; Legault and Wise, 2001).

The results suggest that novelty is detected by the hippocampus and through its connections to the ventral tegmental area, the detection of novelty can elicit dopamine release in the hippocampus, facilitating LTP at the activated synapses (Lisman and Grace, 2005; Shohamy and Adcock, 2010). This idea can be viewed as a neurobiological formulation of the novelty/encoding hypothesis. Thus, the literature on the memory effects of novelty is rather conflicting—on the one hand, human studies produced controversial results, on the other hand, animal studies provided support for the novelty effect. The large variability of novelty manipulations urged the field to define their distinct subtypes.

## The Categorization of Novelty Manipulations

The first attempts at the categorization of novelty were based on the temporal aspect (Berlyne, 1960; Barto et al., 2013). Berlyne distinguished complete novelty, where the organism has never encountered the stimulus before. He also differentiated short-term and long-term novelty, where the stimulus has been encountered before, but not in the last few minutes or the last few days, respectively (Berlyne, 1960). The temporal aspect of novelty is usually not addressed in experimental studies; thus, we can reasonably suppose that most manipulations are aimed to produce complete novelty.

Berlyne also suggested that the amount of novelty in each percept is an important aspect of novelty. He differentiated absolute novelty, which is supposedly a feature that has never been encountered before, while relative novelty is a novel arrangement of familiar features. Berlyne argued that an experienced observer cannot be presented with absolute novelty, as any percept can be related to previous experiences in a meaningful way, as to be only relatively novel. His classic example is that of seeing the tallest man for the first time: this experience may be considered novel, but the man probably only differs significantly in height from other people one has seen (Berlyne, 1960). As absolute novelty may only be experienced by younger children, we can conclude that the current literature studying adult humans and non-human animals is preoccupied with relative novelty.

**Figure 1:**
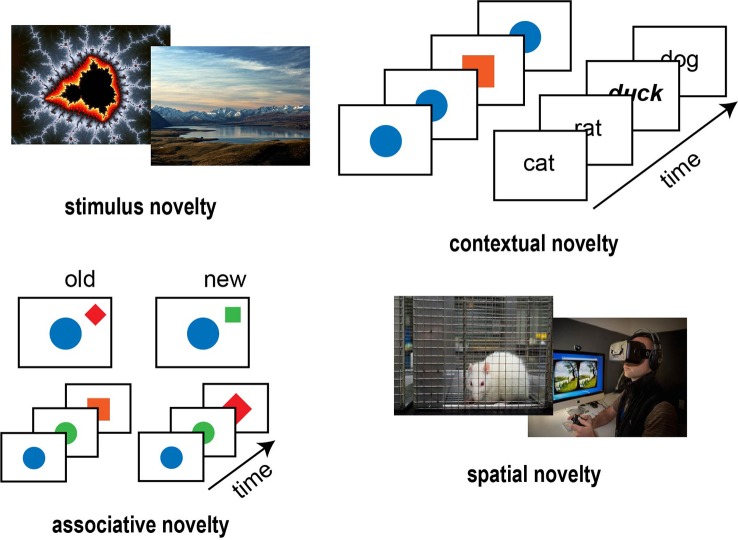
Novelty manipulations in cognitive neuroscience (novmanip.jpg). Stimulus novelty manipulations usually employ stimuli (e.g., fractals, natural scenes) seen only once during the experiment. Contextual novelty manipulations include the oddball paradigm, where a standard stimulus appears most of the time with fewer occurrences of the oddball, and the von Restorff paradigm, where most of the words are presented in the same font while some appear in a different font. In associative novelty manipulations, the participants are familiarized with a spatial or a temporal arrangement of stimuli after which they are presented with a slightly changed arrangement. Spatial novelty manipulations usually involve placing the experimental animal in a novel cage or letting the human participants explore a virtual environment [Original work; Adapted from the University of Melbourne (le.unimelb.edu.au) under CC-BY license].

Although Berlyne’s ideas are certainly of value for the field, the categories he proposed cannot be used to differentiate between the breadth of novelty manipulations appearing in research. Most authors usually distinguish stimulus novelty and contextual novelty (Ranganath and Rainer, 2003), but some also consider associative novelty or even spatial novelty as distinct categories (Nyberg, 2005; Barto et al., 2013; Schomaker and Meeter, 2015; Figure 1). First, stimulus novelty manipulations, probably recognized by most as the archetypal cases of novelty, intend to use stimuli that have never been encountered by the observer before (Barto et al., 2013; Schomaker and Meeter, 2015). A range of different stimuli has been applied in paradigms, where stimulus novelty was manipulated, for example, fractals, simple shapes, natural scenes, and words. Importantly, it is methodically challenging to test memory with these paradigms, since memory performance must be compared across novel vs. well-known stimuli, an inherently biased comparison. This obstacle has been overcome by some paradigms, however, the experimental results obtained with these can be considered mixed on the existence of a clear, stimulus-level memory effect of novelty (Poppenk et al., 2010; Schomaker and Meeter, 2015; Reggev et al., 2017). For example, the classical novelty effect has been shown to result from a source discrimination problem relating to the familiar stimuli (Dobbins et al., 1998; however, see Kormi-Nouri et al., 2005; and Wittmann et al., 2007; for examples of stimulus-level memory effects of novel stimuli).

Second, contextual novelty is defined as a stimulus or an event unexpectedly arising in a given context (Ranganath and Rainer, 2003; Nyberg, 2005). The studies listed under the contextual novelty category mostly utilize some form of the oddball task, where a standard stimulus is presented frequently with the occasional appearance of an oddball stimulus (Ranganath and Rainer, 2003; Barto et al., 2013; Schomaker and Meeter, 2015). Another similar paradigm that falls under the contextual novelty category is the von Restorff paradigm (Nyberg, 2005)—in this paradigm, the subjects learn a list of words, with some words differing in font type or font size (von Restorff, 1933; Karis et al., 1984; Geraci and Manzano, 2010; Schomaker and Meeter, 2015). Contrasting with stimulus novelty, these studies usually show that contextual novelty produces robust benefits on memory performance (Polich, 2007; Barto et al., 2013; Schomaker and Meeter, 2015).

Third, associative novelty is defined as a novel arrangement of familiar stimuli (Nyberg, 2005; Kumaran and Maguire, 2007). Paradigms utilizing associative novelty usually present a spatial or temporal configuration of distinct stimuli in a learning phase and assess the recognition responses of the participants in a test phase, where the arrangements are sometimes shuffled to elicit associative novelty. These experiments are indirectly associated with memory enhancements (Nyberg, 2005; Barto et al., 2013), as associative novelty elicits an increase in the blood-oxygen-level-dependent (BOLD) signal in the hippocampus and the dopaminergic midbrain (Düzel et al., 2003; Schott et al., 2004; Köhler et al., 2005; Kumaran and Maguire, 2006, 2007).

Finally, spatial novelty is distinguished by some authors by their robust effects on memory (Schomaker and Meeter, 2015; Schomaker, 2019). Spatial novelty is a novel spatial environment, and thus spatial novelty manipulations use more complex stimuli than the other categories—in human studies, this involves virtual reality environments (Schomaker et al., 2014b), while in animal studies the subjects are placed in novel cages for example (Jeewajee et al., 2008). These manipulations usually produce marked behavioral manifestations and several physiological correlates of memory enhancements (Schomaker and Meeter, 2015; Schomaker, 2019).

However, this categorization scheme is not without criticism. For example, it has been suggested that contextual and associative novelty manipulations produce memory effects due to unexpectedness, interpreting the reported memory enhancements problematic (Barto et al., 2013). Furthermore, this categorization does not explain the inconsistent findings concerning the effects of novelty on memory performance. Why does one sort of novelty manipulation exert a demonstrable effect on memory, whereas another does not? This question may be answered by the predictive coding framework applied to the functioning of memory systems.

## Predictive Coding and Novelty

The idea that the brain is a predictive system, constantly trying to predict its sensory inputs to ease processing and thus conserve energy, is central to several similar formulations, which we refer to as predictive coding theories (Knill and Pouget, 2004; Friston, 2005, 2010; Clark, 2013). There is ample evidence that such a computational strategy might indeed be utilized in several functional units of the brain (den Ouden et al., 2012; Hyman et al., 2017; de Lange et al., 2018).

The main idea of predictive coding is that the brain constantly uses an inner model to produce predictions about the sensory information that it is likely to encounter. The predictions are compared to the incoming information and only the difference, the prediction error is processed further, which is used to update the inner model so that next time in a similar situation the brain will be able to generate more accurate predictions. The predictive coding framework suggests that the comparisons required to generate prediction errors are made on several different levels in the processing hierarchy of the brain (Knill and Pouget, 2004; Friston, 2005, 2010). The scenario offered by predictive coding is highly relevant for memory research, as one might assume that the inner model, or at least a part of it, could be equated with memory itself and that learning can be considered as the updating mechanism driven by prediction errors (Henson and Gagnepain, 2010; van Kesteren et al., 2012). It is also important to note, however, that prediction errors are also generated on the lower levels of the hierarchy, which may serve veridical perception without influencing information processing at higher levels. This way, prediction errors at lower levels of the processing hierarchy may not necessarily have an impact on learning and memory.

If we take this idea and turn to the memory effects of novelty, however, we find an apparent contradiction. Novelty, by definition, is something that is not contained in the memory systems or the inner model of the observer. As such, it is impossible to accurately predict novelty, therefore it should always generate prediction errors and lead to learning. However, as we have discussed before, this simplistic interpretation is not unequivocally supported by experimental data (Poppenk et al., 2010; Schomaker and Meeter, 2015; Reggev et al., 2017). It could be, that some novelty manipulations employ stimuli that are not deemed important enough by the human information processing system to be encoded and consolidated in long-term memory.

First, we stress that the aspects of our inner models that we can access consciously (which is usually referred to as explicit memory), can only be scarcely detailed (Hobson and Friston, 2012). On the one hand, this assumption is supported by anecdotal evidence from the phenomenology of imagination and lucid dreaming. These processes are thought to be internally generated (using the inner model) and lacking in detail. A thought experiment supports this idea in the case of imagination—when we try to imagine a tiger, we can quickly conclude that minor perceptual details, such as the patterns on the fur, are not stored accurately, as most of us are unable to imagine them precisely (Chater, 2019). People who frequently have lucid dreams also report that during lucid dreaming they are unable to focus on minor visual details of surfaces (Nir and Tononi, 2010), which suggests that this information is not stored in the inner model. On the other hand, theoretical considerations also suggest that the inner model cannot be infinitely detailed, as it would result in a highly unstable system, which requires constant updating, since highly detailed predictions would always produce considerable prediction errors throughout the processing hierarchy (Friston, 2010; Hobson and Friston, 2012). If the inner model is not infinitely detailed, observers may encounter perceptual novelty that does not generate prediction errors driving learning on this level of the hierarchy, simply because the details in question are not stored in those parts of the inner model. This line of thought also suggests that not everything novel will necessarily generate a novel representation in these levels of hierarchy (i.e., in episodic and semantic memory systems). Supposedly, representations enabling veridical perception form on lower-level sensory areas of the brain (den Ouden et al., 2012; de Lange et al., 2018).

Second, we propose that unexpectedness must be of a certain degree to facilitate learning. This idea is most prominently defined in the literature on surprise. However, the use of the term surprise is also somewhat complicated. On one hand, it refers to an emotion that includes a range of physiological, experiential, behavioral and cognitive components, and is elicited by unexpected stimuli or events. On the other hand, surprise can also be defined in an information theory framework as a measure reflecting to what extent is certain sensory data unlikely under a certain belief held by an observer (Friston, 2010; O’Reilly et al., 2013; Seer et al., 2016; Faraji et al., 2018). The physiological and behavioral responses triggered by unexpected stimuli or events are collectively termed the orienting response (Sokolov, 1963; Alexander and Brown, 2019; Reisenzein et al., 2019). This includes directing the appropriate sensory organs of the observer to the source of the discrepancy, skin conductance changes, heart rate deceleration, and pupil dilation. The cognitive processes elicited by unexpectedness are termed the surprise response, according to the cognitive-evolutionary model of surprise (Reisenzein et al., 2019). The surprise response consists of the interruption of the ongoing cognitive processes and a shift of attention to the cause of the discrepancy, which may be accompanied by the conscious experience of surprise, and finally, the learning of the unexpected information. The generation of the surprise response is thought to be based on a comparison between expected and observed inputs, largely paralleling the computation of prediction errors (see above). When the outcome of this comparison exceeds a threshold, a surprise signal is generated, which drives the responses to unexpected events. The idea that a certain threshold needs to be overcome by any given event to elicit these responses is derived from the observation that not any unexpected event readily evokes them (Reisenzein et al., 2019). For example, the surprise response may be elicited by the appearance of a novel coworker, while we may completely ignore a novel item of clothing on a familiar colleague, or a novel item on his/her desk. These examples can be considered cases of stimulus novelty, where the relevance of the stimulus in question differs profoundly just as its effects on cognition. It is conceivable that if we are very interested in clothes or the item in question is the only one on an otherwise empty desk, the surprise response is evoked: motivation, the complexity of the environment or uncertainty may all influence the threshold (i.e., the degree of unexpectedness needed to evoke the surprise response). These ideas fit well with the framework of predictive coding and the suggestion that the degree of prediction errors is central to learning helps to organize the disparate results of novelty manipulations: if unexpectedness elicited by a novelty manipulation reaches a certain threshold, the surprise response is evoked and learning is enhanced.

Here, we propose that to be memorable, novelty must evoke the surprise response, and the reason for the inconsistency in the literature on the memory effects of novelty is that not all novelty manipulations can consistently elicit this phenomenon. In the following sections, we will review the research findings supporting this view.

## Stimulus Novelty: Prediction Errors May Only Serve Veridical Perception

Stimulus novelty is one of the basic categories of novelty manipulations in cognitive neuroscience. Repetition suppression and the novelty N2 are neural responses reliably elicited by stimulus novelty manipulations. Several authors approach stimulus novelty based on its neural effects, or more precisely, the difference between the effects elicited by familiar and novel stimuli (Ranganath and Rainer, 2003; Schomaker and Meeter, 2015). In this view, repetition suppression, which refers to the decreased neural response to repeated stimuli, is the complement of the stimulus novelty effect (i.e., increased neural activation in response to novel stimuli; Ranganath and Rainer, 2003; Nyberg, 2005; Barto et al., 2013). Repetition suppression is a gradual reduction in neural activity in response to repeated items that have been demonstrated in several modalities with numerous methods, ranging from single-cell recordings to functional magnetic resonance imaging (Ranganath and Rainer, 2003; Grill-Spector et al., 2006; Grotheer and Kovács, 2016). A crucial study showed that repetition suppression is modulated by expectation (Summerfield et al., 2008), a finding fitting neatly in the predictive coding scheme, but defying the classic theories of repetition suppression, which postulate that repetition suppression results from inherent properties of neural networks—not subject to higher-level computational effects.

In a predictive coding framework, the activity in the sensory cortices reflects prediction errors, and thus repetition suppression could be a reduction in prediction errors, which may reflect a more accurate prediction of a known stimulus. In this scheme it is conceivable that predictions from higher levels arrive as an inhibition to the neurons exhibiting repetition suppression, hence decreasing the prediction errors that the same input caused before (Friston, 2005; den Ouden et al., 2012; Gotts et al., 2012; Auksztulewicz and Friston, 2016; Grotheer and Kovács, 2016). The finding of Summerfield et al. (2008) that the degree of repetition suppression depends on the probability of repetition, came to be called expectation suppression. Later studies were able to differentiate between repetition suppression and expectation suppression, showing that repetition suppression is an earlier effect which could be followed by expectation suppression (Todorovic and de Lange, 2012; Grotheer and Kovács, 2015). These effects may be epiphenomena caused by the bottom-up traveling of the prediction error, repetition suppression being the expression of a lower level computation and expectation suppression resulting from higher-level prediction errors (Grotheer and Kovács, 2016). Curiously, studies using complex visual stimuli (e. g. fractals, false fonts, natural scenes), the archetypical cases of stimulus novelty, were unable to demonstrate expectation suppression (Kaliukhovich and Vogels, 2011; Kovács et al., 2013; Grotheer and Kovács, 2014). Therefore, repetition suppression seems to depend on the subjects’ expertise with the stimuli (Grotheer and Kovács, 2016), as unfamiliar complex stimuli are harder to predict accurately. We can conclude that although stimulus novelty elicits repetition suppression reliably to enable veridical perception, it does not trigger higher level prediction errors that can elicit expectation suppression or the surprise response and enhance memory.

Stimulus novelty manipulations may also elicit an event-related potential (ERP) component, that is, the anterior N2. The anterior N2 is an ERP, which peaks over frontal regions around 250–300 ms after stimulus presentation in the visual modality and it is the second negative peak following stimulus presentation (Folstein and Van Petten, 2008). It is important to note that the anterior N2 is made up of two different components, one of which is associated with cognitive control and the other one with mismatch detection (Folstein and Van Petten, 2008) or novelty detection (Schomaker and Meeter, 2015). The exact interpretation of the anterior N2 is still debated; however, several authors have proposed that the anterior N2 is an index of novelty processing (Chong et al., 2008; Tarbi et al., 2011; Schomaker et al., 2014a; Schomaker and Meeter, 2015, 2018). The anterior N2 was first observed in oddball paradigms (Folstein and Van Petten, 2008). The simplest of these paradigms is the two-stimulus oddball task, in which the participant is presented with a frequent standard stimulus, requiring no response, and a rare target, which requires a response. Usually, the rare stimulus elicits an anterior N2 (Folstein and Van Petten, 2008), which might appear with a larger amplitude when these stimuli are trial-unique, that is, they appear in only one trial (Breton et al., 1988). Studies with the visual oddball paradigm showed that the appearance and amplitude of the N2 depends on the degree of difference between the perceptual features of frequent and rare stimuli (Courchesne et al., 1975; Thomas and Nelson, 1996; Daffner et al., 2000; Czigler and Balázs, 2005; Barkaszi et al., 2013; Ferrari et al., 2015). A recent study found that expected (vs. unexpected) novel pictures elicited a higher N2, indicating that expectations can enhance the detection of novelty (Schomaker and Meeter, 2018). Based on these findings, several authors proposed that the anterior N2 also reflects low-level prediction errors, which are necessary for veridical perception when there is a considerable change in the incoming visual information (den Ouden et al., 2012; Stefanics et al., 2014; Grotheer and Kovács, 2016).

In sum, two of the most characteristic neural phenomena elicited by stimulus novelty manipulations, the anterior N2, and repetition suppression, seem to reflect low-level prediction errors, which are thought to be necessary for veridical perception (den Ouden et al., 2012; Stefanics et al., 2014; Grotheer and Kovács, 2016). Since veridical perception presumably depends on the statistical model of the visual environment represented in specific neural networks, stimuli that can be processed by the current structure of such networks should not elicit any change on these levels (that is, no visual stimulus for an adult human). Also, the stimuli are probably not unexpected enough after several repetitions to necessitate a change in higher-order networks representing the statistics of the task at hand (i.e., a novel fractal elicits only low-level prediction errors, as its details are processed by the sensory system, however it fails to elicit higher-order prediction errors, as the appearance of another novel fractal is already predictable). Thus, it is conceivable that these prediction errors do not necessitate a lasting update in the inner model, stimulus novelty manipulations do not inherently elicit the surprise response and thus have no inherent effects on memory formation.

On the other hand, several studies seem to suggest that novel stimuli elicit neurophysiological responses associated with memory processes (Bunzeck and Düzel, 2006; Wittmann et al., 2007). Since these studies are mostly built on a neurobiological theory of learning based on animal studies using spatial novelty manipulations, they will be discussed in-depth in a later section of this review, but it is important to note here, that the responses ascribed to novelty in this line of research have been suggested to result from the coinciding unexpectedness (Barto et al., 2013; Schultz, 2016).

## The Surprise Response Is Consistently Elicited by Other Novelty Manipulations

In the next sections, we will examine contextual, associative and spatial novelty manipulations. The studies suggest that these novelty manipulations consistently elicit the surprise response and produce memory-boosting effects (Ranganath and Rainer, 2003; Nyberg, 2005; Schomaker and Meeter, 2015).

### Contextual Novelty

Studies utilizing contextual novelty manipulations probably elicit strong expectations about the presented stimuli, since the standards are very frequent and thus highly probable to appear (Folstein and Van Petten, 2008; Barto et al., 2013; Schomaker and Meeter, 2015). It seems that in these tasks the appearance of the oddballs elicits surprise, as self-reports and the appearance of the surprise response (or orienting response) suggest (Reisenzein et al., 2019).

The P3 ERP component is also evoked in these oddball paradigms (Polich, 2007; Nieuwenhuis et al., 2011) and since the orienting response and the P3 ERP are elicited by highly similar conditions, it has been suggested that the P3 is the neural correlate of the orienting response (Nieuwenhuis et al., 2011). The P3 component can be divided into subcomponents, of which the P3b (or the classical P3 or P300) is generated in the oddball tasks. It has a parieto-central scalp distribution and occurs with 300–400 ms latency after stimulus presentation (Friedman et al., 2001; Polich, 2007; Folstein and Van Petten, 2008; Nieuwenhuis et al., 2011; Schomaker and Meeter, 2015). The amplitude of the P3b is sensitive to the probability of occurrence, the degree of deviance from the standards, the motivational significance of the stimulus and also the degree of attention paid to it (Ranganath and Rainer, 2003; Nieuwenhuis et al., 2011), while the P3b is not sensitive to the physical attributes of the eliciting stimulus (Folstein and Van Petten, 2008; Nieuwenhuis et al., 2011). The relative difference to the standards seems to be more important than the objective or absolute complexity of the stimuli, suggesting that expectancy and not novelty is the central variable determining this response. Also, the P3b tends to habituate as more and more novel stimuli are presented (Nieuwenhuis et al., 2011) and it is conceivable that, as expectation of novelty increases, the elicited prediction errors become insufficient to elicit a surprise response. The von Restorff paradigm could be considered an oddball task with an attached memory test, therefore its effects probably depend on the unexpectedness of the different typesetting, and indeed, deviant words in this task also elicit the P3b (Karis et al., 1984). To sum up, these findings suggest that the P3b is not elicited by novelty *per se*, but it is related to the unexpectedness and presumably the surprise response in studies using contextual novelty manipulations (Schomaker and Meeter, 2015; Reisenzein et al., 2019).

The association between surprise, the orienting response, and the P3 ERP component is close (Nieuwenhuis et al., 2011; Barto et al., 2013; Reisenzein et al., 2019), and thus the neural basis of the P3 is relevant to the neural basis of the surprise response. Some authors proposed that a hippocampal computation is the origin of the P3 (Jeewajee et al., 2008), while others point to parts of the cerebral cortex as the source (Polich, 2007). A concise review of the relevant literature claims that the locus coeruleus (LC) has a major role in generating the P3 (Nieuwenhuis et al., 2011), and the release of norepinephrine in the cortex may be the proximal cause of the P3. The latency of the LC response (~150–200 ms) is in line with the latency of the P3, considering the slow conduction velocity of LC fibers (Nieuwenhuis et al., 2005, 2011). Noradrenaline release is assumed to enhance the signal-to-noise ratio in the affected brain areas, which might be a preparative step to enhance the further analysis of the surprising stimulus, corresponding to the attentional shift in the cognitive-evolutionary model of surprise (Reisenzein et al., 2019). Interestingly, a theory on the function of the P3 suggests that it is a neural correlate of cognitive inhibition (Polich, 2007), which corresponds to the interruption proposed by the cognitive-evolutionary model of surprise. On the other hand, recent studies showed that the LC influences hippocampal memory encoding by releasing dopamine (Kempadoo et al., 2016; Wagatsuma et al., 2018). It seems that the LC elicits the P3 through its effects on the cortex (Nieuwenhuis et al., 2011), while it also alters hippocampal function (Kempadoo et al., 2016; Wagatsuma et al., 2018). Therefore, the hippocampal response could be a correlate, and not the cause of the P3 (Jeewajee et al., 2008). These experiments show that several contextual novelty manipulations can elicit the surprise response and thus can be expected to have memory effects, consistent with the results (Ranganath and Rainer, 2003; Kafkas and Montaldi, 2018).

### Associative and Spatial Novelty

Several studies showed increased hippocampal and midbrain (probably dopaminergic) BOLD activity in response to associative novelty (Düzel et al., 2003; Schott et al., 2004; Köhler et al., 2005; Kumaran and Maguire, 2006, 2007). The memory effects reported in these studies were explained by the influential hippocampal-VTA loop model (Lisman and Grace, 2005). This model organized hippocampal and midbrain responses to novel stimuli into a model, explaining the entry of information into memory.

The main idea is that the CA1 region of the hippocampus compares incoming to stored information and thus computes a novelty signal, in less than 100 ms after stimulus presentation (Ruusuvirta et al., 1995; Brankazk et al., 1996). This signal is transferred to the ventral tegmental area through the downward arc of the hippocampal-VTA loop, which involves the subiculum (Legault and Wise, 2001; Floresco et al., 2003), the nucleus accumbens and the ventral pallidum (Floresco et al., 2001). The ventral tegmental area innervates the hippocampus (Gasbarri et al., 1994a,b) and releases dopamine, which enhances LTP in the hippocampus (Shohamy and Adcock, 2010; Lisman et al., 2011). It is important to note that the studies serving as the basis for this model are mostly animal studies, in which novelty is usually achieved by placing the animals in a novel environment (Floresco et al., 2001; Legault and Wise, 2001).

However, the midbrain dopaminergic responses reported in several studies using associative and stimulus novelty manipulations (Schott et al., 2004, 2006; Bunzeck and Düzel, 2006; Bunzeck et al., 2007; Wittmann et al., 2007, 2008; Krebs et al., 2009) may have been caused by the unexpectedness and not by novelty *per se* (Barto et al., 2013; Schultz, 2016). The reasoning behind this statement builds up from three different clues. First, a study showed that meaningless novel stimuli do not reliably elicit midbrain activations (Stoppel et al., 2009). Second, the study showed directly that dopaminergic midbrain neurons change their firing patterns in response to novel stimuli used light flashes, which did not change during the experiment (Ljungberg et al., 1992), thus they cannot be considered to evoke stimulus novelty. Several authors suggested that it was the unpredictability of the flashes that elicited dopaminergic activity, and novelty is not enough in itself to elicit this response (Barto et al., 2013; Schultz, 2016). Third, it has been shown that hippocampal BOLD responses to novel stimuli quickly habituate as more and more novel items are presented (Murty et al., 2013). These findings suggest that both the hippocampal and the VTA responses are elicited by the unexpectedness and not by novelty.

In an animal study thought to utilize associative novelty, rats have been trained in a maze with simple visual stimuli outside the maze as landmarks (Jenkins et al., 2004). The position of the well-known extra-maze cues was changed on the critical trial and thus familiar stimuli appeared in a novel spatial arrangement, which activated the hippocampus, as indicated by increased c-Fos expression in this region. In another study, it has been shown that the hippocampal theta rhythm is sharply reduced when an animal encounters novelty in its environment, but this is more prominent for unexpected changes in a familiar environment than to a novel environment (Jeewajee et al., 2008). These results seem to suggest that the hippocampal match-mismatch computations compare expectations to actual sensory input, therefore it is also a response to unexpectedness. Since the response pattern of the hippocampus in these studies overlaps with the elicitation requirements of the P3, it has been hypothesized that the reduction in hippocampal theta generates the P3 (Jeewajee et al., 2008). As we discussed, the P3 ERP component is associated with the orienting response, it may be a concomitant of the orienting response (Nieuwenhuis et al., 2011) and as such, a neural correlate of surprise (Barto et al., 2013; Reisenzein et al., 2019).

Spatial novelty as an entirely distinct category is proposed by Schomaker and Meeter, based on the fact that it has a well-described, long-lasting effect on memory (Schomaker and Meeter, 2015). For example, a study showed that viewing novel pictures depicting indoor or outdoor scenes before learning words improves the recollection and the recall of these words (Fenker et al., 2008), while another demonstrated that the exploration of a novel virtual environment before learning produces an increase in the recall of these words (Schomaker et al., 2014b). The studies that established the distinctive memory effect of spatial novelty examined LTP in the hippocampus of rats exploring a novel spatial environment (Straube et al., 2003; Davis et al., 2004; Uzakov et al., 2005) or the BOLD response in the hippocampus of humans performing memory tests involving spatial stimuli (Fenker et al., 2008; Schomaker et al., 2014b). The neural correlates of spatial novelty manipulations are similar to what we have already seen associated with contextual and associative novelty manipulations, thus, it seems reasonable to suggest that the driving force behind them may also be the surprise signal. Space is a central perceptual dimension which is always highly relevant to an organism capable of motion, thus, it is highly probable that unexpected changes in this dimension, like those elicited during the exploration of a novel (let it be real or virtual) environment, generate the surprise signal and drive learning. However, several studies show that these enhancements can be detected even after the exploration of novelty (Fenker et al., 2008; Schomaker et al., 2014b). A possible explanation is that during the exploration of a novel environment surprise signals are generated with a higher frequency and this results in a prolonged enhancement of learning. This would be in line with the “penumbra hypothesis,” which states that increased dopamine availability, which may be a concomitant of the surprise response, benefits memory encoding in general and not just the encoding of the stimulus that elicited its release (Lisman et al., 2011).

In sum, it seems that in associative and spatial novelty manipulations novelty inherently coincides with high unexpectedness. Since the neurophysiological responses associated with these manipulations have been shown to appear in response to unexpected but familiar stimuli, habituate or even fail to appear when supposedly only lower-level prediction errors are elicited by the appearance of a stimulus, the surprise response may provide a more parsimonious explanation for them than a novelty effect. The neurophysiological correlates found in these studies are suggestive of the surprise response—hippocampal activation and the P3 ERP component have been shown to habituate to novelty (Nieuwenhuis et al., 2011; Murty et al., 2013), suggesting that unexpectedness is the relevant stimulus dimension for these responses, as the stimuli are always novel, but the predictability of the occurrence of a novel stimulus increases during the task. It is important to note that the confounding of novelty manipulations by surprise may only be problematic in human studies, while in animal studies novelty and surprise usually coincide because the experimental manipulations are less subtle in this field. Using the same terminology in experimental designs aimed at human participants may lead to confusing results on the memory effects of novelty (Poppenk et al., 2010; Barto et al., 2013; Schomaker and Meeter, 2015; Reggev et al., 2017), as in these experiments the gradual change in unexpectedness may be more impactful for memory formation.

## Conclusion

**Figure 2:**
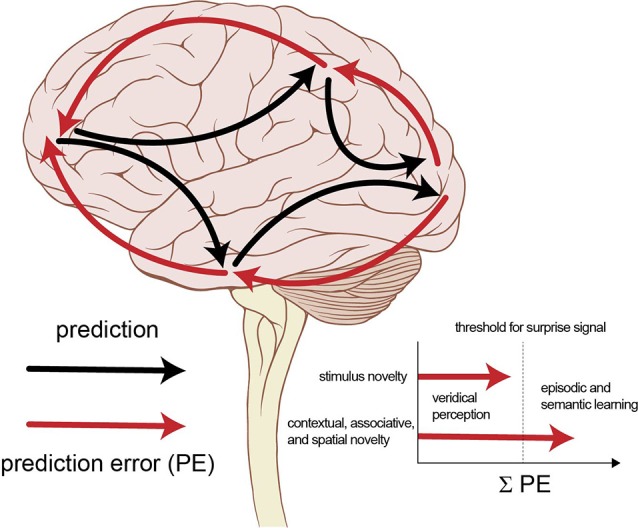
Novelty manipulations and the surprise signal (noveltysurprise.jpg). The brain constantly generates prediction errors as the expectations generated by its inner model are compared to its actual inputs. These prediction errors are summed to judge the necessity of updating the inner model. If the sum of the prediction errors is high enough, it results in the surprise response—the focusing of attention and enhanced learning. Stimulus novelty manipulations usually fail to elicit this response, while it is frequently evoked by contextual, associative and spatial novelty manipulations [Modified figure; Patrick J. Lynch, medical illustrator (https://commons.wikimedia.org/wiki/File:Brain_human_lateral_view.svg), “Brain stem normal human”, added features by R. Reichardt, https://creativecommons.org/licenses/by/2.5/legalcode].

Here, we propose that the memory-enhancing effects of various novelty manipulations used in cognitive neuroscience mostly depend on the degree of unexpectedness (Figure 2). We suggest that inconsistencies of the reported memory effects are in part due to the different degree of unexpectedness induced by the manipulations. Some stimulus novelty manipulations may only elicit perceptual prediction errors in the lower levels of the processing hierarchy, which is required for veridical perception, but the prediction errors generated not always reach the threshold to elicit the surprise response and thus, do not lead to enhanced learning. Contextual, associative and spatial novelty manipulations, however, are often unexpected in addition to being novel and usually elicit greater prediction errors, which result in the updating of the inner model, that is, learning. Spatial novelty could be a special case as it might involve a greater amount of novel information than the other types of novelty, we considered, or due to a special memory effect of spatial information. More work emphasizing the difference between unexpectedness and novelty is needed to organize previous findings meaningfully and to uncover the intricacies of the neurocognitive processes involved in the processing and learning of novel and surprising stimuli or events.

Considering the degree of unexpectedness when it comes to generating a hypothesis about the probable memory effects of a novelty manipulation would be more helpful than judging its similarity to some of the flagship paradigms in a given category. Several recent studies already hinted at this conclusion (Greve et al., 2017; Pine et al., 2018). However, the exact degree of unexpectedness depends on the inner model of the observer, to which the experimenter does not have direct access. Therefore, future studies ought to use an easily quantifiable experimental method, where the unexpectedness of every experimental event can be estimated as a function of the previous events of the task, or design experimental tasks where unexpectedness and novelty are orthogonally varied, to show that unexpectedness drives learning. The degree of unexpectedness is likely to be represented in the brain, but it does not have a single, unambiguous neural correlate (Bach and Dolan, 2012), which further complicates its assessment. Future studies should aim to clarify the neural correlates of surprise, in hopes of providing the means to assess the ability of different experimental manipulations to elicit the surprise response and judge the source of the memory effects more precisely.

Finally, we would like to address a conspicuous omission: this article did not touch upon the effects other variables may have on memory for novel stimuli or events, e.g., top-down control, motivation, stimulus material, retention time, testing method. The motivation of the learner or even her familiarity with the material may have major effects on learning (van Kesteren et al., 2012; Miendlarzewska et al., 2016). These variables may be integrated into the present account as eliciting their effects by changing the threshold for the elicitation of the surprise response, which is modulating the strength of the prediction error required for learning. However, it is important to note that this account of surprise elicited learning only details the starting step of the process: that unexpected stimuli may direct attention and prepare the cognitive system for memory encoding (Lee et al., 2006; Reisenzein et al., 2019). Presumably, depending on how the information to be encoded relates to the information contained in the memory system, its encoding, consolidation, and even retrieval can also be affected by motivation and the novelty of the information itself (van Kesteren et al., 2012; Miendlarzewska et al., 2016).

Another interesting question pertains to the effects of stimulus types and other methodological differences which could also affect memory performance. Could these effects also be integrated into the predictive coding framework? Stimulus material effects may come to be explained through the strength of the prediction errors they elicit, as more complex stimuli may elicit greater prediction errors. Retention time and testing method effects, however, may rely on other neurocognitive processes that occur after initial encoding, although it is admittedly hard to distinguish between these effects with classical paradigms (Cohen et al., 2014). These factors should also be systematically investigated concerning the degree of unexpectedness to draw a complete picture of the memory effects of novelty.

## Author Contributions

RR, BP and PS contributed equally to this work.

## Conflict of Interest

The authors declare that the research was conducted in the absence of any commercial or financial relationships that could be construed as a potential conflict of interest.
